# Alkaline phosphatase combined with γ-glutamyl transferase is an independent predictor of prognosis of hepatocellular carcinoma patients receiving programmed death-1 inhibitors

**DOI:** 10.3389/fimmu.2023.1115706

**Published:** 2023-01-25

**Authors:** Lei Xu, Lin Chen, Bin Zhang, Zhicheng Liu, Qiumeng Liu, Huifang Liang, Yifa Chen, Xiaoping Chen, Chao Leng, Bixiang Zhang

**Affiliations:** ^1^ Hepatic Surgery Center, Tongji Hospital, Tongji Medical College, Huazhong University of Science and Technology, Wuhan, Hubei, China; ^2^ Department of Surgery, Tongji Hospital, Tongji Medical College, Huazhong University of Science and Technology, Wuhan, Hubei, China

**Keywords:** hepatocellular carcinoma, Child-Pugh grade, ALG grade, PD-1 inhibitors, prognosis

## Abstract

**Background:**

Immunotherapy plays an increasingly critical role in the systemic treatment of HCC. This current study aimed to establish a novel prognostic predictor of Programmed death 1 (PD-1) inhibitor therapy in hepatocellular carcinoma (HCC) independent of Child-Pugh grade.

**Methods:**

Our study screened patients with HCC who received PD-1 inhibitors at Tongji Hospital Affiliated to Tongji Medical College of Huazhong University of Science and Technology from January 2018 to December 2020. ALG grade was determined by the patient’s serum ALP and GGT levels before the initiation of PD-1 inhibitors. The endpoints of our study were overall survival (OS) and progression free survival (PFS). Follow-up ended at May 31, 2022.

**Results:**

Eighty- five patients (77 with Child−Pugh grade A, 8 with Child−Pugh grade B at baseline) were enrolled according to the inclusion criteria. Patients with Child−Pugh grade A achieved longer PFS and OS than those with Child−Pugh grade B. Patients with ALG grade 3 at baseline showed worse tumor response and poorer survival, and ALG grade could stratify patients with Child−Pugh grade A into subgroups with significantly different prognosis.

**Conclusions:**

ALG grade, combining ALP and GGT, is a novel and readily available prognostic marker and the predictive effect of ALG grade on patient prognosis is independent of Child−Pugh grade.

## Introduction

Hepatocellular carcinoma (HCC) accounts for approximately 90% of primary liver cancers, and it is one of the leading causes of cancer-related death worldwide (1). Immunotherapy plays an increasingly critical role in the systemic treatment of HCC (2, 3). Previous clinical trials have confirmed the definite antitumor efficacy of programmed death-1(PD-1) inhibitors on HCC (4, 5). The newly released results of RATIONALE-301 (Tislelizumab versus Sorafenib) and LEAP-002 (Lenvatinib plus pembrolizumab versus Lenvatinib) on the European Society for Medical Oncology in 2022 highlighted the key role of PD-1 inhibitors monotherapy in the treatment of HCC (NCT03412773, NCT03713593). PD-1 inhibitors are emerging as a critical part of guideline-recommended first-line therapy for advanced hepatocellular carcinoma (NCT03298451) (6).

Preserved liver function is the premise of various treatments for HCC, and it is an important predictor of superior prognosis (7). Previous research confirmed that better liver function was positively associated with the prognosis of patients after hepatectomy (8), local therapy (9, 10) and systemic therapy (11, 12). Child−Pugh grade is extensively used to evaluate patient liver function when systemic therapy is recommended, and most patients are in Child−Pugh grade A, so it is necessary to develop other liver function evaluation systems to further stratify them into different groups according to their prognosis.

Alkaline phosphatase (ALP) and γ-glutamyl transferase (GGT) are readily available as part of serum liver function tests, and they are elevated in the presence of cholestasis or liver parenchymal damage. Previous studies suggested that elevated serum ALP and GGT were independent risk factors for the prognosis of HCC patients (13, 14). Other scholars have found that ALP and GGT have the potential to be used as biomarkers to predict the cancer risk and mortality of all comers (15, 16). Few studies have explored the role of ALP and GGT in the prognosis of HCC patients receiving programmed death 1(PD-1) inhibitors.

In this retrospective study, we analyzed the data of patients with HCC who received PD-1 inhibitors to explore the prognostic effect of ALP and GGT in HCC.

## Methods

### Patients

Our study screened patients with HCC who received PD-1 inhibitors at Tongji Hospital Affiliated to Tongji Medical College of Huazhong University of Science and Technology from January 2018 to December 2020. To further explore the risk factors associated with PD-1 inhibitor therapy for hepatocellular carcinoma, we enrolled patients receiving PD-1 inhibitors monotherapy without targeted drug therapy. The inclusion criteria were as follows: 1) confirmed HCC diagnosed pathologically or clinically; 2) regular treatment with PD-1 inhibitors no less than 2 cycles; 3) no history of molecularly targeted drug therapy; 4) case data sufficient for efficacy evaluation; 5) at least 18 months of follow-up since the start of the PD-1 inhibitors. We collected the patients’ basic information, treatment history, and relevant fluid examination and imaging results. The current study was approved by the Medical Ethics Committee of Tongji Hospital Affiliated to Tongji Medical College of Huazhong University of Science and Technology (TJ-IRB20220936).

### Treatment evaluation

Follow-up ended at May 31, 2022. The endpoints of our study were overall survival (OS) and progression free survival (PFS). Overall survival was defined as the interval between the initiation of PD-1 inhibitor treatment and death or the last follow-up, and PFS was defined as the interval from the initiation of PD-1 inhibitor therapy to confirmed tumor progression or the last follow-up. Tumor response was evaluated according to the immune Response Evaluation Criteria in Solid Tumors (iRECIST) (17). Tumor response was divided into four grades: immune complete response (iCR), immune partial response (iPR), immune unconfirmed progressive disease (iUPD), immune confirmed progressive disease (iCPD) and immune stable disease (iSD). Tumor assessment was performed every 6-12 weeks. Liver cirrhosis was confirmed according to the diagnostic criteria issued by the Chinese Medical Association (18).

### Alkaline phosphatase & gamma-glutamyl transferase (ALG) grade

ALG grade was determined by the patient’s serum ALP and GGT levels before the initiation of PD-1 inhibitors. The normal ranges of serum ALP and GGT are 40-130 U/L and 10-71 U/L, respectively, in Tongji Hospital. Both serum ALP and GGT higher than normal values were recorded as ALG grade 3, serum ALP or GGT higher than normal was defined as ALG grade 2, serum ALP and GGT within normal ranges were recorded as ALG grade 1.

### Statistics

Data analysis was performed by SPSS 26.0 and GraphPad Prism 8 software. Continuous variables were compared by independent samples t-test or Mann−Whitney U test, while categorical variables were compared by chi-square test and Fisher’s exact probability test. Spearman rank correlation analysis was used to test the correlation between rank variables. Kaplan−Meier survival analysis and log-rank test were used to calculate survival curves and compare differences. Univariate and multivariate cox regression models were performed to explore the risk factors of overall survival and progression free survival. P < 0.05 was considered statistically significant.

## Results

### Patient characteristics

A total of 395 HCC patients received PD-1 inhibitors at Tongji Hospital during the study period, and 85 patients (76 males and 9 females) were enrolled according to the inclusion criteria. Among the enrolled patients, forty-four received radical hepatectomy before immunotherapy. Different brands of PD-1 inhibitors were used in enrolled patients (sintilimab: n=50, pembrolizumab: n=9, camrelizumab: n=18, toripalimab: n=8), and none of them received molecularly targeted drugs. Seventy−seven of the enrolled patients (77/85, 90.6%) lived with Child−Pugh grade A at baseline, and the other 8 patients (8/85, 9.4%) lived with grade B. Patients were classified as ALG grade 1 (n=33), ALG grade 2 (n=26), and ALG grade 3 (n=26) according to serum ALP and GGT levels before the first PD-1 inhibitor treatment. The patients’ baseline characteristics were summarized in **Table 1**. It is worth noting that there was significant difference in the proportion of macrovascular invasion among patients with different ALG grades. Spearman rank correlation analysis showed that ALG grade was positively correlated with tumor macrovascular invasion (r=0.297, P=0.006).

**Table 1:** Demographic characteristics of 85 patients with HCC who received immunotherapy.

	ALG Grade 1 (N=33)	ALG Grade 2 (N=26)	ALG Grade 3 (N=26)	P value
Age(mean ± SD)	54.64 ± 2.09	53.35 ± 2.03	51.38 ± 2.05	0.534
Sex(N/%)				0.553
Male	28/84.8	24/92.3	24/92.3	
Female	5/15.2	2/7.7	2/7.7	
Drinking history (N/%)				0.551
Yes	6/18.2	4/15.4	7/26.9	
No	27/81.8	22/84.6	19/73.1	
Smoking history(N/%)				0.936
Yes	14/42.4	10/38.5	10/38.5	
No	19/57.6	16/61.5	16/61.5	
Treatment history before immunotherapy				
Radical hepatectomy				0.235
Yes	22/66.7	12/46.2	13/50.0	
No	11/33.3	14/53.8	13/50.0	
TACE				0.620
Yes	19/57.6	12/46.2	15/57.7	
No	14/42.4	14/53.8	11/42.3	
Microwave ablation				0.072
Yes	6/18.2	2/7.7	1/3.8	
No	27/81.8	24/92.3	25/96.2	
Chemotherapy				0.740
Yes	4/12.1	2/7.7	4/15.4	
No	29.87.9	24/92.3	22/84.6	
Treatment history after immunotherapy				
Microwave ablation				0.203
Yes	4/12.1	1/3.8	1/3.8	
No	29/87.9	25/96.2	25/96.2	
TACE				0.343
Yes	12/36.4	5/19.2	7/26.9	
No	21/63.6	21/80.8	19/73.1	
Radiotherapy				0.183
Yes	1/3.0	1/3.8	3/11.5	
No	30/97.0	25/96.2	23/88.5	
Chemotherapy				0.971
Yes	5/15.2	2/7.7	4/15.4	
No	28/84.8	24/92.3	22/84.6	
HBV infection				0.754
Yes	30/90.9	23/88.5	23/88.5	
No	3/9.1	3/11.5	3/11.5	
AFP on first diagnose(N/%)				0.721
>200ng/mL	14/42.4	13/50.0	12/46.1	
≤200ng/mL	19/57.6	13/50.0	14/53.9	
AFP before immunotherapy(N/%)				0.279
>200ng/mL	11/33.3	12/42.3	12/42.3	
≤200ng/mL	22/66.7	14/57.7	14/57.7	
Liver cirrhosis				0.368
Present	13/39.4	13/50.0	15/57.7	
Absent	20/60.6	13/50.0	11/42.3	
Tumor number				0.171
Single	22/66.7	15/57.7	11/42.3	
Multiple	11/33.3	11/42.3	15/57.7	
Tumor size				0.073
≥ 5cm	20/60.6	21/80.8	22/84.6	
< 5cm	13/39.4	5/19.2	4/15.4	
Macrovascular invasion				0.020*
Present	2/6.1	7/26.9	9/34.6	
Absent	31/93.9	19/73.1	17/65.1	
Distant metastasis				0.845
Present	4/12.1	2/7.7	3/11.5	
Absent	29.87.9	24/92.3	23/88.5	
Child-Pugh grade before immunotherapy(N/%)				0.100
A	32/97.0	24/92.3	21/80.8	
B	1/3.0	2/7.7	5/19.2	
BCLC stage on first diagnosis(N/%)				0.959
A	10/30.3	7/26.9	6/23.1	
B	15/45.5	11/42.3	12/46.1	
C	8/24.2	8/30.8	8/30.8	
BCLC stage before immunotherapy(N/%)				0.521
0	6/18.2	8/30.8	4/15.4	
A	7/21.2	3/11.5	3/11.5	
B	12/36.4	9/34.6	8/30.8	
C	8/24.2	6/23.1	11/42.3	
PD-1 inhibitors				0.276
Sintilimab	18/54.5	18/69.2	14/53.8	
Pembrolizumab	6/18.2	2/7.7	1/3.8	
Camrelizumab	6/18.2	3/11.5	9/34.6	
Toripalimab	3/9.1	3/11.5	2/7.7	
Immunotherapy course (mean ± SD)	6.55 ± 0.69	5.31 ± 0.88	4.85 ± 0.60	0.224

*: P<0.05; TACE, Transcatheter arterial chemoembolization; BCLC, Barcelona Clinic Liver Cancer; AFP, alpha fetoprotein. HBV, Hepatitis B virus.

At the end of follow-up, 36 patients suffered tumor progression, 23 patients died during follow-up, and none of them died from complications related to immunotherapy.

### Tumor response

Among total 85 patients, five (5/85, 5.9%) achieved iPR, forty-five (45/85, 52.9%) patients achieved stable disease, and others (35/85, 41.2%) suffered tumor progression. The objective response rate (ORR) and disease control rate (DCR) were 5.9% (5/85) and 58.8% (50/85) in our cohort. Subgroup analysis showed that the DCR in patients with ALG grade 3 was significantly lower than that in patients with ALG grades 1, but there was no significant difference in DCR between patients with ALG grades 1 and 2. Patients with different ALG grades achieved similar ORRs. **Table 2** summarized the tumor response of patients with different ALG grades.

**Table 2:** Tumor response of patients with different grade of ALG.

	ALG Grade 1 (N=33)	ALG Grade 2 (N=26)	ALG Grade 3 (N=26)	P value
iPR (N/%)	3/9.1	1/3.8	1/3.8	0.568
iSD (N/%)	21/63.6	15/57.7	9/34.6	0.078
iPD (N/%)	9/27.3	10/38.5	16/61.5	0.030*
ORR (%)	9.1	3.6	3.8	0.909
DCR (%)	72.7	61.5	38.5	0.028*

*: P<0.05; iPR, immune partial response; iSD, immune stable disease iPD, immune progressive disease; ORR, Objective response rate; DCR, Disease control rate.

### Survival analysis

**Figure 1:**
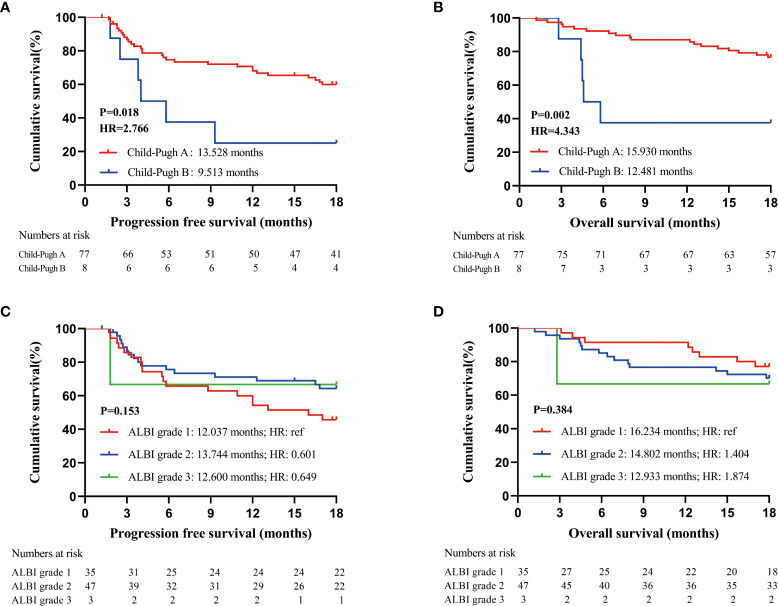
Prognostic analysis of patients receiving PD-1 inhibitors; **(A)**. PFS of all enrolled patients according to Child-Pugh grade at baseline; **(B)**. OS of all enrolled patients according to Child-Pugh grade at baseline; **(C)**. PFS of all enrolled patients according to ALBI grade at baseline; **(D)**. OS of all enrolled patients according to ALBI grade at baseline.

The mean PFS and OS of patients in the cohort were 12.986 (95% CI: 11.574-14.398) months and 15.326 (95% CI: 14.231-16.421) months, respectively. Child−Pugh grade and Albumin-Bilirubin (ALBI) grade are the most commonly used grading system for liver function evaluation. Both PFS and OS of patients with Child−Pugh grade A (PFS: 13.528 months, 95% CI: 12.087-14.968; OS: 15.930 months, 95% CI: 14.909-16.950) were significantly superior than those of patients with Child−Pugh grade B in our study (PFS: 9.513 months, 95% CI: 4.927-14.908; OS: 12.481 months, 95% CI: 9.952-15.009) (PFS: P=0.018, OS: P=0.002) (**Figures 1A, B**). PFS and OS were similar in patients with different ALBI grades in our study (**Figures 1C, D**).

**Figure 2:**
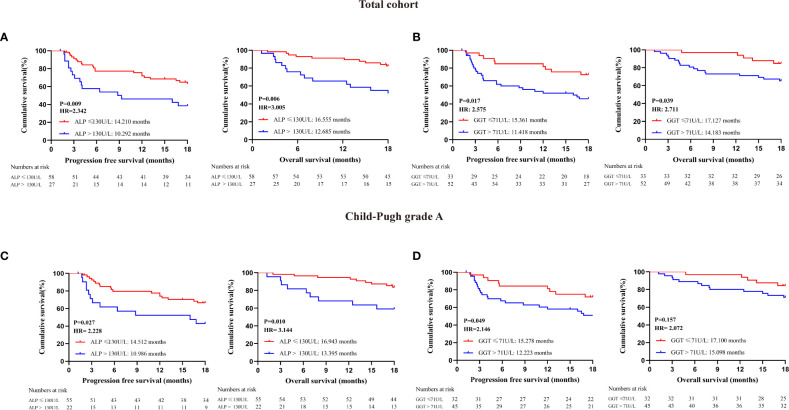
Prognostic analysis of patients receiving PD-1 inhibitors; **(A)** Survival analysis of all enrolled patients with different serum ALP levels at baseline: **(B)** Survival analysis of all enrolled patients with different serum GGT levels at baseline; **(C)** Survival analysis of patients with Child-Pugh grade A according to serum ALP levels at baseline; **(D)** Survival analysis of patients with Child-Pugh grade A according to serum GGT levels at baseline.

**Figure 3:**
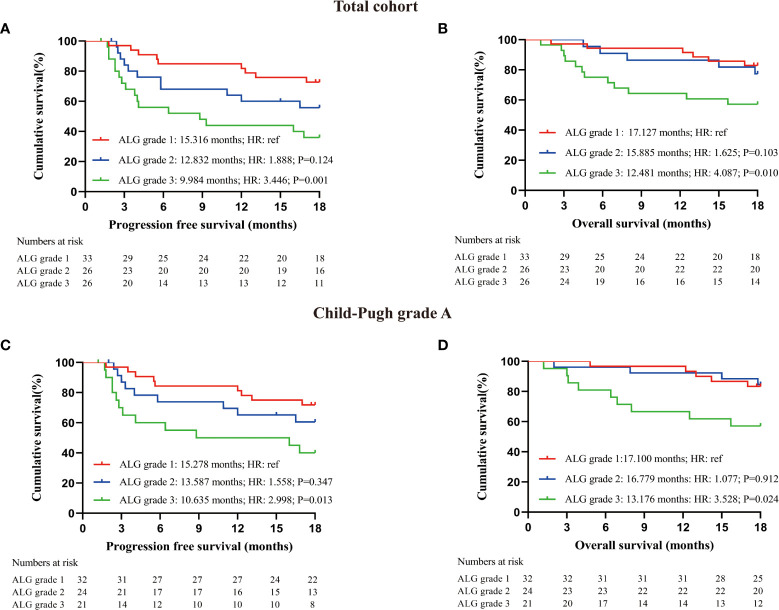
Prognostic analysis of patients receiving PD-1 inhibitors; **(A)** PFS of all enrolled patients with different ALG grade at baseline; **(B)** OS of all enrolled patients with different ALG grade at baseline; **(C)** PFS of patients with Child-Pugh grade A according to serum ALG grade at baseline; **(D)** OS of patients with Child-Pugh grade A according to serum ALG grade at baseline. Log-rank test was used to compare the prognostic differences between groups.

To explore more effective predictors, we conducted analysis according to the serum ALP and GGT values before the first dose of PD-1 inhibitors. Firstly, in the whole cohort, patients with normal serum ALP and GGT before anti-PD-1 treatment achieved longer PFS and OS than those with elevated serum ALP and GGT (**Figures 2A, B**). Subsequently, we found that ALP and GGT could further stratify the prognosis of patients with the same Child−Pugh grade. Elevated serum ALP and GGT predicted poor prognosis in patients with Child−Pugh grade A (**Figures 2C, D**). We further combined ALP and GGT as a new indicator to enhance prediction accuracy and ease of use. Both the PFS and OS of patients with ALG grade 1 (PFS: 15.316 months, 95CI: 13.640-17.082; OS: 17.127 months, 95CI: 16.240-18.015) were significantly longer than those of patients with ALG grade 3 (PFS: 9.984 months, 95CI: 7.203-12.765; OS: 12.481 months, 95CI: 9.952-15.009) (PFS: P=0.001, OS: P=0.010). Patients with ALG grade 2 (PFS: 12.832 months, 95CI: 10.263-15.401; OS: 15.885 months, 95CI: 14.066-17.703) also had longer PFS and OS than those with ALG grade 3, but the difference was not statistically significant (PFS: P=0.124, OS: P=0.103) (**Figures 3A, B**). The ALG grade could also stratify patients with Child−Pugh grade A into subgroups with significantly different prognosis (**Figures 3C, D**).

Moreover, there was no correlation between ALG grade and Child−Pugh grade in our study (P=0.100), indicating that the prognostic predictive effect of ALG grade is independent of Child−Pugh grade. Receiver operating characteristic (ROC) curves were generated to compare the predictive effects of indicators. The area under ROC curve of ALG grade, Child−Pugh grade, serum ALP and GGT levels at baseline for PFS was 0.657, 0.563, 0.620, and 0.610, respectively. The area under ROC curve of ALG grade, Child-Pugh grade, serum ALP and GGT levels at baseline for OS was 0.672, 0.585, 0.640, and 0.617, respectively. ALG grade is better than Child−Pugh grade in predicting the prognosis of patients, and has no correlation with Child−Pugh grade.

### Prognostic factors

We established cox regression models to explore risk factors of PFS and OS (**Tables 3**, **4**). Univariate analysis showed that ALG grade 3 (HR: 3.446, 95% CI: 1.519-7.819), ALP>130 U/L (HR: 2.342, 95% CI: 1.212-4.526), GGT>71 U/L (HR: 2.575, 95% CI: 1.209-5.485) and Child−Pugh grade B before immunotherapy (HR: 2.766, 95% CI: 1.143-6.692) were associated with PFS of patients receiving PD-1 inhibitors. Meanwhile, liver cirrhosis (HR: 3.500, 95% CI: 1.379-8.885), ALG grade 3 (HR: 4.087, 95% CI: 1.437-11.621), ALP>130 U/L (HR: 3.005, 95% CI: 1.324-6.822), GGT>71U/L (HR: 2.711, 95% CI: 1.006-7.307), AFP≥200 ng/mL (HR: 2.353, 95% CI: 1.018-5.440) and Child−Pugh grade B before immunotherapy (HR: 4.343, 95% CI: 1.595-11.827) were associated with the OS of patients. Multivariate Cox regression proved that ALG grade 3 (HR: 3.446, 95% CI: 1.519-7.819) was an independent risk factor of PFS, liver cirrhosis (HR: 3.514, 95% CI: 1.383-8.928) and Child−Pugh grade B before immunotherapy (HR: 4.518, 95% CI: 1.652-12.537) were independent risk factors of OS.

**Table 3:** Cox regression analysis of PFS for patients receiving immunotherapy.

Variables	Univariate analysis				Multivariate analysis			
	HR	95%CI		P	HR	95%CI		P
		Lower	Upper			Lower	Upper	
Sex (Male/Female)	5.135	0.730	37.512	0.107				
Age≥60	0.433	0.180	1.041	0.062				
Drinking history	1.623	0.781	3.370	0.194				
Smoking history	1.326	0.689	2.551	0.399				
Radical hepatectomy	0.989	0.514	1.902	0.972				
BCLC stage on first diagnose	0.996	0.480	2.067	0.992				
liver cirrhosis	1.370	0.712	2.637	0.346				
Child-Pugh grade B before immunotherapy	2.766	1.143	6.692	0.024*				
AFP≥200ng/mL on first diagnose	0.809	0.272	2.407	0.703				
APRI≥2 before immunotherapy	0.934	0.286	3.051	0.910				
FIB-4≥3.25 before immunotherapy	0.963	0.471	1.967	0.917				
Child-Pugh grade B before immunotherapy	2.766	1.143	6.692	0.024				
AFP≥200ng/mL before immunotherapy	1.782	0.855	3.713	0.123				
SII>300 before immunotherapy	1.092	0.561	2.124	0.796				
NLR>1.9 before immunotherapy	1.720	0.606	4.879	0.308				
PLR>130U/L before immunotherapy	0.937	0.450	1.951	0.861				
ALG grade								
1	Ref.	Ref.	Ref.	Ref.				
2	1.888	0.782	4.560	0.158				
3	3.446	1.519	7.819	0.003**	3.446	1.519	7.819	0.003**
GGT>71U/L before immunotherapy	2.575	1.209	5.485	0.014*				
ALB>35g/L before immunotherapy	0.809	0.272	2.407	0.703				
ALP>130U/L before immunotherapy	2.342	1.212	4.526	0.011*				

*: P<0.05, **: P<0.01. HR, Hazard ratio; BCLC, Barcelona Clinic Liver Cancer; AFP, alpha fetoprotein; APRI, Aspartate aminotransferase/Platelet ratio index; FIB-4, Fibrosis 4 score; SII, systemic immune-inflammation index; NLR, Neutrophil to Lymphocyte ratio; PLR, Platelet to Lymphocyte ratio; ALP, Alkaline phosphatase; GGT, gamma-glutamyl transferase; ALB, Albumin.

**Table 4:** Cox regression analysis of Overall survival for patients receiving immunotherapy.

Variables	Univariate analysis				Multivariate analysis			
	HR	95%CI		P	HR	95%CI		P
		Lower	Upper			Lower	Upper	
Sex(Male/Female)	1.313	0.308	5.559	0.713				
Age≥60	0.356	0.106	1.197	0.095				
Drinking history	0.811	0.276	2.385	0.704				
Smoking history	1.443	0.636	3.271	0.380				
Radical hepatectomy	1.020	0.450	2.312	0.962				
BCLC B/C stage on first diagnose	1.122	0.442	2.846	0.809				
liver cirrhosis	3.500	1.379	8.885	0.008**	3.514	1.383	8.928	0.008*
Child-Pugh grade B at first diagnose	2.137	0.725	6.300	0.169				
AFP≥200ng/mL at first diagnose	1.240	0.526	2.920	0.623				
Child-Pugh grade B before immunotherapy	4.343	1.595	11.827	0.004**	4.518	1.652	12.537	0.003**
AFP≥200ng/mL before immunotherapy	2.353	1.018	5.440	0.045*				
APRI≥2 before immunotherapy	1.051	0.246	4.496	0.947				
FIB-4≥3.25 before immunotherapy	1.263	0.530	3.013	0.598				
SII>300 before immunotherapy	1.700	0.713	4.504	0.231				
NLR>1.9 before immunotherapy	1.269	0.297	5.341	0.748				
PLR>130U/L before immunotherapy	1.439	0.604	3.431	0.412				
ALG grade								
1	Ref.	Ref.	Ref.	Ref.				
2	1.625	0.496	5.326	0.423				
3	4.087	1.437	11.621	0.008*				
GGT>71U/L before immunotherapy	2.711	1.006	7.307	0.049*				
ALB>35g/L before immunotherapy	0.626	0.271	1.447	0.273				
ALP>130U/L before immunotherapy	3.005	1.324	6.822	0.009**				

*: P<0.05, **:P<0.01. HR, Hazard ratio; BCLC, Barcelona Clinic Liver Cancer; AFP, alpha fetoprotein; APRI, Aspartate aminotransferase/Platelet ratio index; FIB-4, Fibrosis 4 score; SII, systemic immune-inflammation index; NLR, Neutrophil to Lymphocyte ratio; PLR, Platelet to Lymphocyte ratio; ALP, Alkaline phosphatase; GGT, gamma-glutamyl transferase; ALB, Albumin.

## Discussion

Our study confirmed that elevated baseline serum ALP and GGT levels, as indicators of liver parenchymal damage, were prognostic risk factors of HCC patients receiving PD-1 inhibitors, and the interference of molecularly targeted drugs was excluded according to the inclusion criteria. We also reported that ALG grade, a new combination of ALP and GGT, was a superior predictor of prognosis for the first time, and ALG grade can further stratify patients with Child−Pugh grade A into subgroups with different prognoses.

PD-1 inhibitor therapy for HCC is an emerging systemic therapy, and liver function at baseline is a confirmed prognostic factor. Child−Pugh grade is one of the most common clinical indicators for evaluating liver reserve capacity, and studies have confirmed that the prognosis of patients with Child−Pugh Grade B were worse than that of patients with Child−Pugh Grade A (19), and Albumin-Bilirubin (ALBI) grade is another common liver function scoring method that can replace Child−Pugh score (20). Our study found that Child-Pugh grade, rather than ALBI grade was associated with patient prognosis. Since most patients receiving systemic therapy have good liver function reserve in the early stage, Child−Pugh grade and ALBI grade cannot accurately stratify the prognosis of such patients, we explored other indicators in serum liver function tests. In our study, both PFS and OS of patients with normal ALP and GGT levels at baseline were superior than those of patients with elevated ALP and GGT receiving PD-1 inhibitors. Previous studies supported that elevated ALP and GGT were associated with poor survival of patients receiving liver resection (21, 22), liver transplantation (23, 24), local therapy (25, 26) and molecular targeted therapy (27, 28). This was consistent with our findings in anti-PD-1 therapy, and it is noteworthy that serum ALP and GGT levels could stratify patients with Child−Pugh grade A, enhancing the accuracy of prediction in the current study. Cox regression further confirmed the results of survival analysis. Liver function is also a critical factor in the long-term survival of patients, and this is similar to our findings. We further found that ALG grade 3 was also a prognostic risk factor for patients with Child−Pugh grade A, and there was no significant correlation between Child−Pugh grade and ALG grade. ALG grade is a reliable prognostic factor independent of Child−Pugh grade, but ALG grade 3 was an independent risk factor for PFS but not for OS in our study. The reason of this result may be: 1. most of the patients enrolled have not yet reached the clinical endpoint, only 23 patients died during follow-up; 2. the follow-up time (18 months) was relatively short; 3. Statistical differences should not be the only factor in the validity of prognostic indicators, ALG grade 3 was risk factor for PFS which has confirmed the prognostic effect of ALG grade to a certain extent.

Serum ALP and GGT are common diagnostic indicators for liver diseases. As early as 1985, elevated GGT was systematically reported to be associated with the occurrence of liver cancer (29). Drug-induced liver injury is common in patients with advanced HCC, elevated serum ALP and GGT levels hinder recovery from drug-induced liver injury (30, 31), and the concentrations of ALP and GGT in the bile duct can be used as indicators to quantify bile duct injury (32). GGT increases the level of intracellular glutathione, which can promote tumor cells to maintain balance in reactive oxygen species and avoid cell death through redox pathways during pro-oxidant therapy (33), GGT-positive tumors possess stronger proliferative ability and drug resistance (34, 35). Researchers have demonstrated that granulocyte colony-stimulating factor (G-CSF) upregulates GGT1 and enhances the immunosuppressive function of myeloid-derived suppressor cells, and GGT inhibitors can alleviate tumor immunosuppression and the tumor-promoting effect of G-CSF (36). Alkaline phosphatase is ubiquitous expressed in human body, which has four types: intestinal ALP, placental ALP, germ cell ALP and single tissue non-specific ALP (37). Previous studies have found that placental ALP is highly expressed in HCC cell lines, and this type of ALP can transform cells with specific survival advantages to malignant transformation (38). Tumor cells with ALP overexpression are more likely to form immunogenic cold tumors, and targeted killing of ALP-overexpressing tumor cells can convert immunogenic cold tumors to hot (39). This further confirms the plausibility of our finding that GGT and ALP are adverse long-term prognostic factors for HCC patients receiving PD-1 inhibitors.

There are still some limitations in the current study: First, this is a single-center, retrospective clinical study, and the level of evidence for the findings is not strong enough; Second, the number of participants in the study was relatively small, sufficient sample size and follow-up time can more realistically analyze the prognosis of patients; Third, systemic treatments are diverse for HCC patients, and our study only excluded the interference of molecular targeted therapy on risk factor analysis. Mutation and amplification of oncogenes and tumor suppressor genes also affect the long-term prognosis of patients with HCC (40). Multicenter, prospective clinical trials are still needed to further explore the role of ALG grade in the prognosis of patients with HCC receiving PD-1 inhibitors.

Our study indicated that ALG grade 3 at baseline predicted poor prognosis of patients with HCC receiving PD-1 inhibitors. ALG grade, combining ALP and GGT, is a novel and readily available prognostic marker and the predictive effect of ALG grade on patient prognosis is independent of Child−Pugh grade.

## Data availability statement

The original contributions presented in the study are included in the article/supplementary materials. Further inquiries can be directed to the corresponding authors.

## Ethics statement

Our study was approved by the Medical Ethics Committee of Tongji Hospital Affiliated to Tongji Medical College of Huazhong University of Science and Technology (TJ-IRB20220936). Written informed consent for participation was not required for this study in accordance with the national legislation and the institutional requirements. Written informed consent was not obtained from the individual(s) for the publication of any potentially identifiable images or data included in this article.

## Author contributions

LX and CL wrote the paper; LX, BinZ, and ZL collected and analyzed the clinical data;QL and HL added part of clinical information; XC revised the manuscript; CL and BixZ designed this subject. All authors contributed to the article and approved the submitted version.
