# Erratum: Alkaline phosphatase combined with γ-glutamyl transferase is an independent predictor of prognosis of hepatocellular carcinoma patients receiving programmed death-1 inhibitors

**DOI:** 10.3389/fimmu.2023.1177261

**Published:** 2023-03-08

**Authors:** Frontiers Production Office

**Affiliations:** Frontiers Media SA, Lausanne, Switzerland

**Keywords:** hepatocellular carcinoma, Child-Pugh grade, ALG grade, PD-1 inhibitors, prognosis

Due to a production error, an author name was incorrectly spelled as “Zhichen Liu”. The correct spelling is “Zhicheng Liu”. The publisher apologizes for this mistake.

The original version of this article has been updated.

